# Global mismatch between greenhouse gas emissions and the burden of climate change

**DOI:** 10.1038/srep20281

**Published:** 2016-02-05

**Authors:** Glenn Althor, James E. M. Watson, Richard A. Fuller

**Affiliations:** 1 School of Geography, Planning and Environmental Management, University of Queensland, Queensland, 4072, Australia; 2 Wildlife Conservation Society, Global Conservation Program, 2300 Southern Boulevard, Bronx, NY 10460-1068, USA; 3 School of Biological Sciences, University of Queensland, Queensland, 4072, Australia

## Abstract

Countries export much of the harm created by their greenhouse gas (GHG) emissions because the Earth’s atmosphere intermixes globally. Yet, the extent to which this leads to inequity between GHG emitters and those impacted by the resulting climate change depends on the distribution of climate vulnerability. Here, we determine empirically the relationship between countries’ GHG emissions and their vulnerability to negative effects of climate change. In line with the results of other studies, we find an enormous global inequality where 20 of the 36 highest emitting countries are among the least vulnerable to negative impacts of future climate change. Conversely, 11 of the 17 countries with low or moderate GHG emissions, are acutely vulnerable to negative impacts of climate change. In 2010, only 28 (16%) countries had an equitable balance between emissions and vulnerability. Moreover, future emissions scenarios show that this inequality will significantly worsen by 2030. Many countries are manifestly free riders causing others to bear a climate change burden, which acts as a disincentive for them to mitigate their emissions. It is time that this persistent and worsening climate inequity is resolved, and for the largest emitting countries to act on their commitment of common but differentiated responsibilities.

The current generation is the first to feel the effects of anthropogenic climate change^1^,^2^. Despite their well-known harmful impacts to the world’s climate system^1^,^3^, greenhouse gases (GHG) are deliberately emitted by countries to drive economic growth and enhance human wellbeing^4^. Spatially localised environmental issues, such as city air pollution^5^, may result from high GHG emissions, but the most damaging and long lasting consequence, that of global climate change^6^, is not constrained within the border of the emitting country^1^. Rather, by polluting the Earth’s atmosphere with GHG emissions through fossil fuel combustion, deforestation and agricultural activities, emitting countries are degrading the world’s climate system, a common resource shared by all biodiversity, including people^7^,^8^.

Because the impacts of GHG emissions can be felt beyond a country’s border, and the impacts of climate change on countries are highly variable, there is potential for some emitters to contribute more or less to the causes of climate change than is proportionate to their vulnerability to its effects^9^,^10^,^11^. This inequity has not gone unnoticed in international climate negotiations or global reporting^1^,^3^. As far back as 1992, the United Nations Framework Convention on Climate Change (UNFCCC) committed to the principle of “common but differentiated responsibilities”, in which countries have a common responsibility in reducing GHG emissions, but historic emissions and differences in current development levels mean that countries have different levels of emissions reduction obligations^9^. Both of the previous IPCC Assessment Reports have acknowledged the inequity in the causes and effects of climate change^1^,^12^ although operationalising the principle has proved difficult^13^. This is primarily because developing and developed countries continue to disagree over the extent of each other’s responsibilities^13^,^14^. One major impediment to resolving such debates is a poor quantitative understanding of the magnitude of the global inequity in emissions and impacts. ‘Free rider’ countries contribute disproportionately to global GHG emissions with only limited vulnerability to the effects of the resulting climate change, while ‘forced rider’ countries are most vulnerable to climate change but have contributed little to its genesis^15^,^16^. This is an issue of environmental equity on a truly global scale^17^.

Here, we measure the current pattern of global climate change equity, and assess whether the situation will improve or worsen by 2030, using data on GHG emissions^17^ and newly available national climate change vulnerability assessments^18^. We address the lack of a contemporary, qualitative assessment of global climate equity that incorporates key variables. Previous studies have been limited to CO_2_ emissions datasets, omitting the most potent and long lasting GHGs^1^,^6^,^16^, and used vulnerability variables that do not capture the complexity of climate change threats, and cannot be forecasted. Here, we use the most recently available datasets based on comprehensive national vulnerability assessments and comprehensive GHG emissions data to produce an easily replicable snapshot of the relationship between countries’ GHG emissions and their vulnerability to the negative effects of climate change^17^,^18^, and forecast this to 2030. We employ economic metrics, the Gini and Robin Hood coefficients^19^, to quantify the present level of equity in GHG emissions. Only through a proper empirical understanding of the pattern of climate equity now, and how it will change in the near future, can signatories of the UNFCCC make meaningful progress toward resolving the inequity in the burden of climate change impacts.

## Results

**Figure 1:**
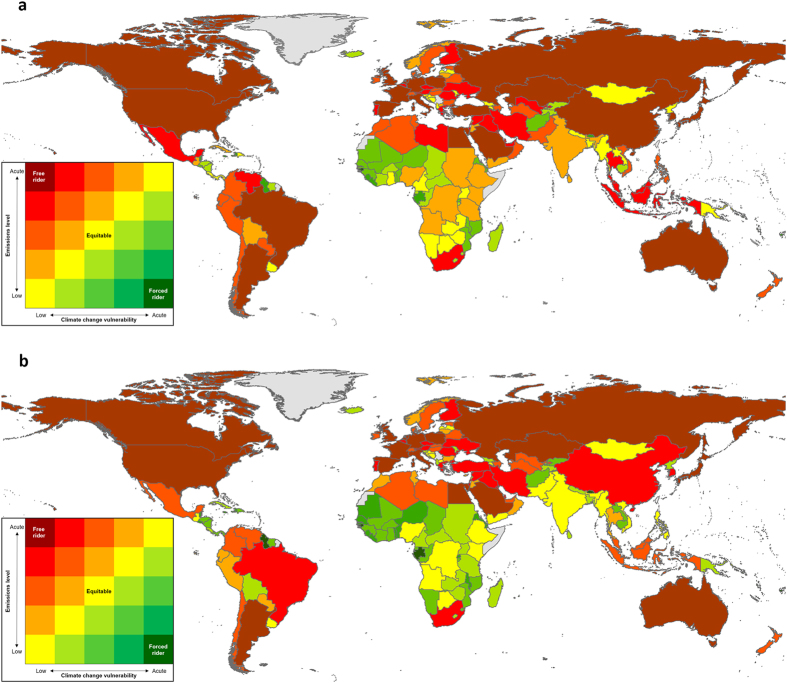
Global inequity in the responsibility for climate change and the burden of its impacts. (**a**) Climate change equity for 2010. (**b**) Climate change equity for 2030. Countries with emissions in the highest quintile and vulnerability in the lowest quintile are shown in dark red (the climate free riders), and those countries with emissions in the lowest quintile and vulnerability in the highest quintile are shown in dark green (the climate forced riders). Intermediate levels of equity are shown in graduating colours, with countries in yellow producing GHG emissions concomitant with their vulnerability to the resulting climate change. Data deficient countries are shown as grey. Maps generated using ESRI ArcGIS^36^.

**Figure 2:**
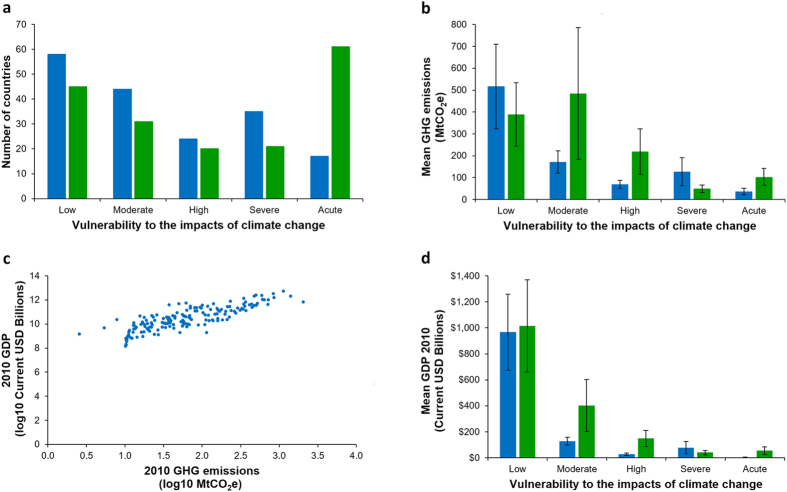
Vulnerability to climate change, mean GHG emissions, and mean GDP. (**a**) Number of countries in each climate change vulnerability category, derived from DARA vulnerability data^18^, for 2010 (blue bars) and 2030 (green bars). (**b**) Mean GHG emissions for 2010, derived from CAIT GHG emissions data^17^, shown in CO_2_ equivalent units and climate vulnerability categories for 2010 (blue bars, with standard error) and 2030 (green bars, with standard error). (**c**) GDP shown in current US$ (in billions), derived from the World Bank GDP 2010 data^28^, and 2010 GHG emissions. (**d**) Mean GDP for 2010 shown in current US$ (in billions) and climate change vulnerability for 2010 (blue bars, with standard error) and 2030 (green bars, with standard error).

Greenhouse gas emissions are spread highly unevenly across the world’s countries (Fig. 1), with the top ten GHG emitting countries generating >60% of total emissions, and three countries, China (21.1%), the United States of America (14.1%) and India (5.2%) being by far the largest contributors. A Gini coefficient of 80.9 indicated extreme inequality in the distribution of emissions among countries, given that the index can only vary between 0 (perfectly even responsibility) and 100 (one country responsible for all emissions)^19^. A Robin Hood index of 64 indicated that 64% of GHG emissions would need to be redistributed to achieve an even distribution among countries^19^. Vulnerability to the impacts of climate change was also unevenly spread among countries, with 17 countries acutely vulnerable to climate change impacts in 2010 (Fig. 2). The majority of these were island countries located in the Atlantic, Pacific and Indian oceans (n = 7, 35.3%) and African countries (n = 8, 47%). By 2030 the number of acutely vulnerable countries is predicted to rise dramatically (n = 62; Fig. 2), and the majority of these will again be island (n = 20, 32.8%) and African (n = 27, 44.2%) countries.

Countries least vulnerable to the impacts of climate change were generally the highest GHG emitters, and conversely those most vulnerable to climate change were the least responsible for its genesis. This inequity held true for both 2010 and 2030, with a negative relationship between emissions and climate vulnerability in both years (2010: ρ = −0.4, n = 175, p = 0; 2030: ρ = −0.37, n = 175, p = 0). The only exception is in 2030, where countries acutely vulnerable to climate change will have slightly higher average emissions than those in the severe category (2030: severe = 48.83 mtCO_2_e, acute = 103.13 mtCO_2_e).

In 2010, of the 179 countries assessed, 28 (15.6%) were in the same quintile for GHG emissions and vulnerability to the negative impacts of climate change. This indicates that their vulnerability to climate change approximately matched their relative contribution to its genesis (Fig. 1). Ninety countries (50.3%) had GHG emissions in a higher quintile than their 2010 climate vulnerability, and 20 (11.2%) countries were free riders, with GHG emissions in the highest quintile and climate vulnerability in the lowest quintile (Fig. 1; see Supplementary Table S4 online). Sixty-one (34%) countries had GHG emissions in a lower quintile than their climate vulnerability, and six (3.4%) countries were forced riders, with GHG emissions in the lowest quintile and climate vulnerability in the highest quintile (Comoros, Gambia, Guinea-Bissau, São Tomé and Príncipe, Solomon Islands and Vanuatu; see Supplementary Table S4 online).

By 2030, climate change inequity will rise further, with an increase in the proportion of countries that are forced riders (n = 20; 11.2%), but fewer free riders (n = 16; 8.9%) and equitable countries (n = 23; 12.8%; see Supplementary Table S4 online). Free riders are typically located in the world’s sub-tropical and temperate regions, while forced riders are frequently located in tropical regions (Fig. 1).

Greenhouse gas emissions were positively correlated with GDP (2010: ρ = 0.84, n = 175, p = 0; Fig. 2c), while climate vulnerability declined with increasing GDP (2010: ρ = −0.69, n = 175, p = 0; 2030: ρ = −0.65, n = 175, p = 0; Fig. 2d). Our analysis considers the absolute contribution of each country to climate change, but we also examined climate change equity in per capita terms to provide a more complete picture of emissions responsibilities. The patterns were broadly similar, with, for example, Australia, Russia and the United States of America remaining free riders (see Supplementary Fig. S3 online). However, several populous major emitters (e.g. United Kingdom, China, and Brazil) were no longer categorised as free riders.

## Discussion

Climate change inequity is globally pervasive, and correlated with economic output. Some countries, such as China and the United States of America, are in a win-win position of achieving economic growth through fossil fuel use with few consequences from the resulting climate change, while many other, mostly Island and African, countries suffer low economic growth and severe, negative climate change impacts (see Supplementary Table S4 online). The beneficiaries of this climate inequity have few incentives to meaningfully reduce or halt their GHG emissions. Despite many of the broad issues around climate equity being well known^1^, well-funded global mechanisms that are being implemented still do not exist. This has serious consequences for our ability to slow the rate of climate change, and reduce the wellbeing implications for forced rider countries.

There are several global policy frameworks currently being debated that could address elements of the problem. The Paris Agreement^20^, secured at the 21^st^ UNFCCC Conference of the Parties (COP21), for example, sets an ambitious target of limiting global warming to 1.5°C above preindustrial levels. However, the 160 indicative nationally determined contributions (INDCs) pledges submitted by signatories to the UNFCCC prior to COP21^21^, indicate that current targets for GHG emissions are unlikely to limit warming to below 2°C^22^ With no binding agreement established at COP21 for INDCs, there is no clear indication of how successful the Paris Agreement will be^20^. Addressing GHG emissions is clearly an important first step in ensuring the burden of climate change is not amplified in the future. However, the historic commitment to GHG emissions reduction by key free riders has been slow. Only 50 countries ratified the previous Doha Amendment to the Kyoto protocol, which did not include key free riders such as the United States and Russia^23^. Furthermore, some countries have actually backtracked on their commitments to emissions reductions (e.g. Canada and Australia)^24^,^25^.

Likewise, the Paris Agreement calls for urgent and adequate financing of US$100 billion per year by 2020 for climate mitigation and adaptation through the Financial Mechanism of the Convention (FMC)^20^. However, there is no legally binding mechanism under which parties are responsible for providing this funding. History suggests such funding goals are not always met. For example, the Green Climate Fund (GCF) was established in 2010 under the UNFCCC to mobilise funding support for the least developed countries that are most vulnerable to climate change, yet it remains poorly funded, with only US$10.2 billion received in pledges by November 2015^26^. Addressing these issues around climate funding will play a critical role in addressing climate inequity^27^.

## Conclusion

It is clear climate change inequity must be addressed. If the commitment to the principle of common but differentiated responsibilities that was widely accepted early on in the UNFCCC is to be acted upon, member states now need to do much more to hold climate free riders to account. To ensure equitable outcomes from climate negotiations, there needs to be a meaningful mobilization of policies, such as the Paris Agreement, that achieve national level emissions reductions, and to ensure the vulnerable forced-rider countries are able to adapt rapidly to climate change. The provisioning of these policy mechanisms will require a distribution of resources and responsibilities and we believe our results provide one way to understand where these responsibilities lie. The Paris Agreement may be a significant step forward in global climate negotiations. However, as the Agreement’s key policies are yet to be realized, member states have both an exceptional opportunity and a moral impetus to use these results to address climate change equity in a meaningful manner.

## Methods

We quantified climate change equity, defined as the distribution of climate change benefits and burdens, using data from two publicly available datasets and national GDP data. National level data sets suffer from some weaknesses such as a lack of accounting for sub-national variability and scaling. Nonetheless, they are still highly useful as global metrics as they provide aggregated assessments at the national level, which is the most meaningful for international policy negotiations.

We extracted data on national vulnerability to the negative impacts of climate change from DARA’s Climate Vulnerability Monitor (CVM)^18^. The CVM uses 22 climate vulnerability indicators across four impact areas (Environmental Disasters, Habitat Change, Health Impact, and Industry Stress) to evaluate the vulnerability of 184 countries to climate change impacts for the years 2010 and 2030. Each of the 22 indicators is individually aggregated from various data sources and models and then combined to determine a country’s overall climate vulnerability, measured by impact to share of GDP and mortality (as these impacts are comparable across the wide range of countries). The CVM calculates vulnerability projections for 2030 using human population growth, mortality and GDP predictions. The CVM uses five vulnerability categories (low, medium, high, severe and acute) which are determined using a mean absolute standard deviation method^18^. The CVM categories do not of course capture the full complexity of national climate vulnerability, as capturing this would require an impractical degree of data. However, we consider the 22 indicators used by the CVM as capturing a high enough level of complexity to provide a meaningful approximation of national vulnerability.

Data on GHG emissions (by countries) were exported from the World Resource Institute’s (WRI) Climate Analysis Indicators Tool (CAIT)^17^, a database of national and international GHG emissions derived from multiple sources. The CAIT data set compiles data for the six main GHG gasses (carbon dioxide (CO_2_), methane (CH_4_), nitrous oxide (N_2_O), hydrofluorocarbons (HFCs), perfluorocarbons (PFCs) and sulfur hexafluoride (SF_6_)) from 185 countries over the period from 1990–2012. We used the 2010 data for this study to match the CVM vulnerability data. The WRI compiled GHG data from UNFCCC reports and complemented with data from several NGO sources^17^, including emissions data from six major sectors (Land Use Change & Forestry, Energy, Industrial Processes, Agriculture, Waste, and International Bunkers) and several subsectors. The CAIT data set reports at the national level, however we extrapolated per capita emissions results by dividing data by 2010 and 2030 population data from the World Bank^28^ (see Supplementary Table S4 online).

We excluded the ten countries (Cook Islands, Federated States of Micronesia, Marshall Islands, Montenegro, Nauru, Niue, Saint Kitts & Nevis, Serbia, Somalia and Taiwan) with data missing in any dataset, and 179 remained for analysis. In addition, there were also insufficient data available for many of the world’s island and archipelagic countries. Given the negligible GHG emissions and high climate change vulnerability of such countries, the majority are highly likely to qualify as climate forced riders^29^,^30^ and as such, we expect that climate forced rider countries are likely underrepresented in our results. National GDP (measured in Current US$) was extracted from the World Bank Group^28^, who measure GDP as the gross value of all resident producers in an economy plus taxes.

We created a Lorenz curve to represent the variation of GHG emissions among countries using the CAIT dataset, and calculated the Gini index to measure inequity in GHG emissions among countries, and the Robin hood index to measure how much of the total global emissions would have to be redistributed to achieve equity among countries (see Supplementary Fig. S2 online).

We compared the CAIT GHG data and the CVM vulnerability data both in 2010 and 2030 to assess whether the most heavily polluting countries were also those least vulnerable to the negative effects of climate change. We divided the CAIT GHG emissions into quintiles, matching the CVM data, to enhance comparability between the datasets and enable visualisation of climate equity in the recent past (2010) and near future (2030). We placed the emissions quintiles on a scale between the highest (acute emissions) and the lowest (low emissions) emitting countries. We also tested the correlations between GHG emissions and GDP against vulnerability to climate change by treating vulnerability categories as ordinal data and undertaking spearman’s rho tests using R statistical software^31^. R has a computational limitation for p-values lower than 2.2e-16, as such, where values this small were reported we wrote “p = 0”. Additionally, we counted countries in each CVM category and compared them between each time period.

In common with other studies of inequity in climate change^32^, we used terminology from the economics literature to define ‘free riders’ and ‘forced riders’^33^, recognising that a strict definition of these terms often applies only to situations where one agent’s use of a resource does not directly incur a cost to another agent. We define climate free riders as those countries in the ‘acute’ GHG emissions quintile and the ‘low’ vulnerability category, as they disproportionately receive benefits from climate change (via the national wellbeing generated by GHG emissions) but pay few costs in the sense they are the least vulnerable to negative climate change effects^34^,^35^. Conversely, we define climate forced riders as those countries that fall within the ‘acute’ vulnerability category and the ‘low’ GHG emissions quintile, as they are the most susceptible to the negative consequences of climate change but receive the least benefits. Those countries that we define as equitable, fall in the same emissions quintile and vulnerability category (for example, low emissions quintile, low vulnerability category), as their emissions benefits are concomitant with their climate change burden.

## Additional Information

**How to cite this article**: Althor, G. *et al.* Global mismatch between greenhouse gas emissions and the burden of climate change. *Sci. Rep.* **6**, 20281; doi: 10.1038/srep20281 (2016).

## Supplementary Material

Supplementary Information

Supplementary Information

## References

[b1] Ipcc. Climate Change 2014: Impacts, Adaptation, and Vulnerability. Part A: Global and Sectoral Aspects. Contribution of Working Group II to the Fifth Assessment Report of the Intergovernmental Panel on Climate Change [ Field C. B., Barros V. R., Dokken D. J., Mach K. J., Mastrandrea M. D., Bilir T. E., Chatterjee M., Ebi K. L., Estrada Y. O., Genova R. C., Girma B., Kissel E. S., Levy A. N., MacCracken S., Mastrandrea P. R., and White L. L. (eds.)]. (Cambridge University Press, 2014).

[b2] Ipcc. Climate Change 2014: Impacts, Adaptation, and Vulnerability. Part B: Regional Aspects. Contribution of Working Group II to the Fifth Assessment Report of the Intergovernmental Panel on Climate Change [ Barros V. R., Field C. B., Dokken D. J., Mastrandrea M. D., Mach K. J., Bilir T. E., Chatterjee M., Ebi K. L., Estrada Y. O., Genova R. C., Girma B., Kissel E. S., Levy A. N., MacCracken S., Mastrandrea P. R. & White L. L. (eds.)]. (Cambridge University Press, 2014).

[b3] United Nations Framework Convention on Climate Change. Text of the Convention. (United Nations Framework Convention on Climate Change, 1992).

[b4] Jorgenson A. K. Economic development and the carbon intensity of human well-being. Nature Clim. Change 4, 186–189 (2014).

[b5] Sheehan P., Cheng E., English A. & Sun F. China’s response to the air pollution shock. Nature Clim. Change 4, 306–309 (2014).

[b6] Montzka S. A., Dlugokencky E. J. & Butler J. H. Non-CO2 greenhouse gases and climate change. Nature 476, 43–50 (2011). 21814274 10.1038/nature10322

[b7] Betts R. Comparing apples with oranges. Nature Reports Climate Change 2, 7–8, doi: 10.1038/climate.2007.74 (2008).

[b8] Stocker B. D. *et al.* Multiple greenhouse-gas feedbacks from the land biosphere under future climate change scenarios. Nature Clim. Change 3, 666–672 (2013).

[b9] Cazorla M. & Toman M. in Climate Change Economics and Policy: An RFF Anthology Ch. 23, 235 (RFF Press, 2001).

[b10] Kjellstrom T., Kovats R. S., Lloyd S. J., Holt T. & Tol R. S. J. The Direct Impact of Climate Change on Regional Labor Productivity. Arch. Environ. Occup. Health 64, 217–227 (2009). 20007118 10.1080/19338240903352776

[b11] Trenberth K. Changes in precipitation with climate change. Climate Research 47, 123–138 (2011).

[b12] Ipcc. Climate Change 2007: Impacts, Adaptation and Vulnerability : Working Group ii Contribution to the Fourth Assessment Report of the Ipcc [ Parry M. L., Canziani O. F., Palutikof J. P., Van Der Linden P. J. & Hanson C. E. (Eds)]. (Cambridge University Press, 2007).

[b13] Ostrom E. A polycentric approach for coping with climate change. Ann. Econ. Finance 15, 71–108 (2014).

[b14] Cole D. H. Advantages of a polycentric approach to climate change policy. Nature Clim. Change 5, 114–118 (2015).

[b15] Rao N. D. International and intranational equity in sharing climate change mitigation burdens. Int. Environ. Agreem.-P. 14, 129–146 (2014).

[b16] Füssel H.-M. How inequitable is the global distribution of responsibility, capability, and vulnerability to climate change: A comprehensive indicator-based assessment. Global Environmental Change 20, 597–611 (2010).

[b17] World Resources Institute, C *limate Analysis Indicators Tool: WRI’s Climate Data Explorer.* (2014) Available at: http://cait.wri.org/historic. (Date of access: 31/05/2015).

[b18] DARA. *Methodological Documentation for the Climate Vulnerability Monitor.* (DARA, 2012).

[b19] Coulter P. B. Measuring Inequality: A Methodological Handbook. (Westview Press, 1989).

[b20] UNFCCC. Conference of the Parties to the United Nations Framework Convention on Climate Change. (UNFCCC, 2015).

[b21] UNFCCC, *INDCs as communicated by Parties*. (2015) Available at: http://www4.unfccc.int/submissions/indc/Submission%20Pages/submissions.aspx. (Date of access:) 17/12/15).

[b22] Jackson R. B. *et al.* Reaching peak emissions. *Nature Clim. Change.* Advance online publication, (2015). Available at: http://www.nature.com/nclimate/journal/vaop/ncurrent/full/nclimate2892.html. (Date of access: 17/12/2015)

[b23] Fekete H. *et al.* Analysis of Current Greenhouse Gas Emission Trends. (Climate Analytics, 2013).

[b24] Pizer W. A. & Yates A. J. Terminating links between emission trading programs. J Environ Econ Manag 71, 142–159 (2015).

[b25] Harrison K. A Tale of Two Taxes: The Fate of Environmental Tax Reform in Canada. Review of Policy Research 29, 383–407 (2012).

[b26] Green Climate Fund, *Background.* (2015) Available at: http://www.gcfund.org/about/the-fund.html. (Date of access: 02/11/2015).

[b27] Pickering J., Jotzo F. & Wood P. J. Splitting the difference: can limited coordination achieve a fair distribution of the global climate financing effort ? Environ Polit 15, 4 (2015).

[b28] World Bank Group, *World DataBank.* (2015) Available at: http://databank.worldbank.org/data/views/reports/tableview.aspx. (Date of access: 31/05/2015).

[b29] Nurse L. A. *et al.* in Climate Change 2014: Impacts, Adaptation, and Vulnerability. Part B: Regional Aspects. Contribution of Working Group II to the Fifth Assessment Report of the Intergovernmental Panel of Climate Change (eds Barros V. R. *et al.* .) Ch. 29, 1613–1654 (Cambridge University Press, 2014).

[b30] Widlansky M. J. *et al.* Changes in South Pacific rainfall bands in a warming climate. Nature Clim. Change 3, 417–423 (2013).

[b31] Core Team R. (2013). *R: A language and environment for statistical computing.* R Foundation for Statistical Computing, Vienna, Austria. URL http://www.R-project.org/.

[b32] Otto F. E. L., Frame D. J., Otto A. & Allen M. R. Embracing uncertainty in climate change policy. Nature Clim. Change 5, 917–920 (2015).

[b33] Ahnlid A. Free or Forced Riders ? : Small States in the International Political Economy: The Example of Sweden. Cooperation and Conflict 27, 241–276 (1992).

[b34] Keohane R. O. The Global Politics of Climate Change: Challenge for Political Science. PS: Political Science & Politics 48, 19–26 (2015).

[b35] Kennedy M. & Basu B. An analysis of the climate change architecture. Renewable and Sustainable Energy Reviews 34, 185–193 (2014).

[b36] ESRI (2011). *ArcGIS Desktop*. Environmental Systems Research Institute, Redlands, CA. URL http://www.arcgis.com/.

