# Supplementary material for: Global mismatch between greenhouse gas emissions and the burden of climate change
Source: Sci Rep. 2016 Feb 5;6:20281. doi: 10.1038/srep20281 (PMC4742864; doi:10.1038/srep20281)
Supplement: Supplementary Information [file srep20281-s1.docx]

**Supplementary information for:**

**Global mismatch between greenhouse gas emissions and the burden of climate change**

Glenn Althor^1^, James E.M. Watson^1,2^, Richard A. Fuller^3^

^1^School of Geography, Planning and Environmental Management, University of Queensland, Queensland, 4072, Australia.

^2^Wildlife Conservation Society, Global Conservation Program, 2300 Southern Boulevard, Bronx, NY 10460-1068, USA.

^3^School of Biological Sciences, University of Queensland, Queensland, 4072, Australia.

**S1 Overview**

This document provides supplementary information not provided in the main text of the article Global mismatch between greenhouse gas emissions and the burden of climate change. S2 shows the climate equity Lorenz curve with Gini and Robin hood indices. S3 shows the results of performing the same analyses provided in the Methods of the main text, but uses per-capita emissions. S4 presents a table of data used for analyses.

**S2 Summary of Lorenz curve**

As detailed in the Methods of the main text, equity indices were created to measure climate inequity. We created a Lorenz curve (Supplementary Fig. S2) which shows the equity of the distribution of GHG emissions data for the year 2010[^1^](#_ENREF_1). The blue line represents the hypothetical line of equality where countries would reside if GHG emissions were divided with perfect equity between all countries. The red line represents the Lorenz curve and denotes actual GHG emissions equity for the year 2010. The distance between the blue and red lines represents GHG emissions inequity and is quantified by the Gini (80.9) and Robin Hood indices (64). As such, the curve and both indices show that the current distribution of GHG emissions is highly inequitable.

**S3 A summary of per capita GHG emissions and national vulnerability**

We conducted separate analyses to those reported in the Methods section of the main text, using per capita GHG emissions. We created a similar set of maps to those in the main text for per capita emissions by dividing emissions by national populations [^2^](#_ENREF_2) (Supplementary Fig. S3). Per capita emissions are shown for years 2010 (Supplementary Fig. S3a) and 2030 (Supplementary Fig. S3b). Maps generated using ESRI ArcGIS [^3^](#_ENREF_3).The patterns we found here are broadly similar to those in the main text (Supplementary Fig. S3c). For example, Australia, Russia and the United States of America remain as free riders. However, several populous major emitters (e.g. United Kingdom, China, Brazil) were no longer categorised as free riders.

**S4 Summary of data**

A table of all countries, their level of vulnerability at 2010 and 2030, GHG emissions, GDP at 2010 and population at 2010 and 2030 is presented (Supplementary Table S4).

**Supplementary Figure S2**


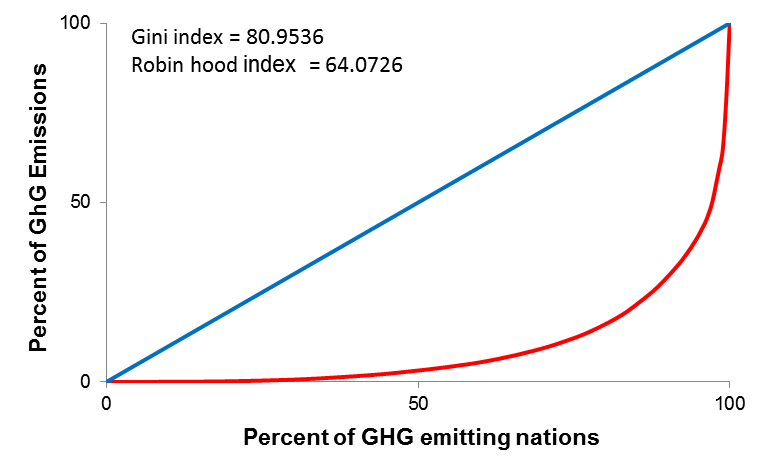


**Supplementary Figure S3**

**a**

**
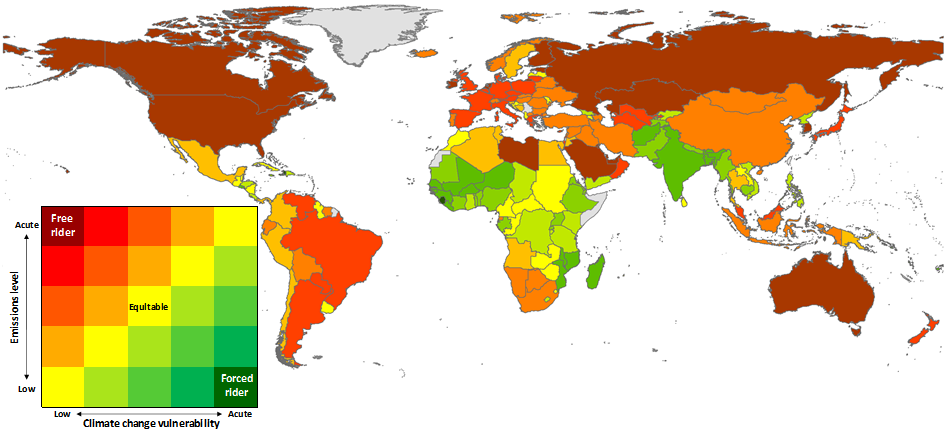
**

**b**
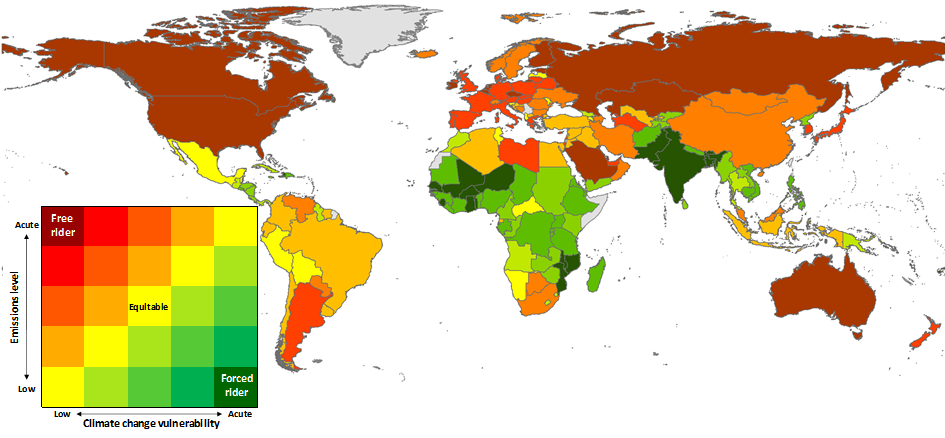


**c**


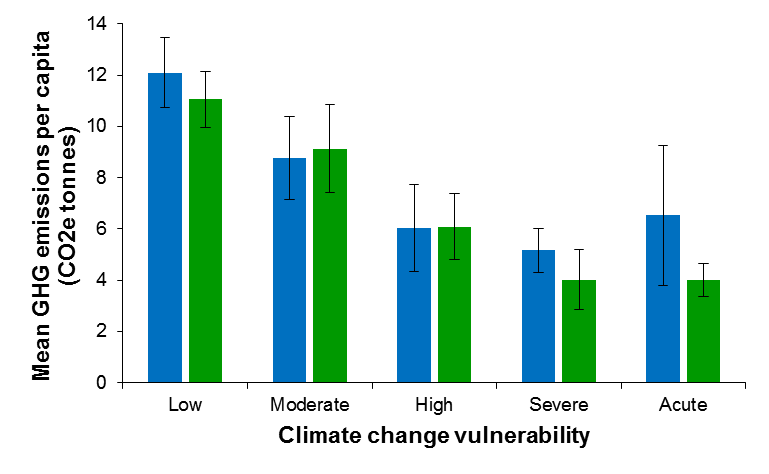


1 Coulter, P. B. *Measuring inequality: a methodological handbook*. (Westview Press, 1989).

2 World Bank Group. World DataBank. (2015). <http://databank.worldbank.org/data/views/reports/tableview.aspx>.

3 ESRI ArcGIS. Release 10. *Redlands, CA: Environmental Systems Research Institute* (2011).
